# Glucansucrase (mutant) enzymes from *Lactobacillus reuteri* 180 efficiently transglucosylate *Stevia* component rebaudioside A, resulting in a superior taste

**DOI:** 10.1038/s41598-018-19622-5

**Published:** 2018-01-24

**Authors:** Evelien M. te Poele, Tim Devlamynck, Manuel Jäger, Gerrit J. Gerwig, Davy Van de Walle, Koen Dewettinck, Anna K. H. Hirsch, Johannis P. Kamerling, Wim Soetaert, Lubbert Dijkhuizen

**Affiliations:** 1 Microbial Physiology, Groningen Biomolecular Sciences and Biotechnology Institute (GBB), University of Groningen, Nijenborgh 7, 9747 AG Groningen, The Netherlands; 2 Centre for Industrial Biotechnology and Biocatalysis, Department of Biochemical and Microbial Technology, Faculty of Bioscience Engineering, Ghent University, Coupure Links 653, 9000 Ghent, Belgium; 3 Stratingh Institute for Chemistry, University of Groningen, Nijenborgh 7, 9747 AG Groningen, The Netherlands; 4 NMR Spectroscopy, Bijvoet Center for Biomolecular Research, Utrecht University, Padualaan 8, 3584 CH Utrecht, The Netherlands; 5 Laboratory of Food Technology and Engineering, Faculty of Bioscience Engineering, Ghent University, Coupure Links 653, 9000 Ghent, Belgium; 6 Present Address: CarbExplore Research BV, Zernikepark 12, 9747 AN Groningen, The Netherlands

## Abstract

Steviol glycosides from the leaves of the plant *Stevia rebaudiana* are high-potency natural sweeteners but suffer from a lingering bitterness. The *Lactobacillus reuteri* 180 wild-type glucansucrase Gtf180-ΔN, and in particular its Q1140E-mutant, efficiently α-glucosylated rebaudioside A (RebA), using sucrose as donor substrate. Structural analysis of the products by MALDI-TOF mass spectrometry, methylation analysis and NMR spectroscopy showed that both enzymes exclusively glucosylate the Glc(β1→C-19 residue of RebA, with the initial formation of an (α1→6) linkage. Docking of RebA in the active site of the enzyme revealed that only the steviol C-19 β-D-glucosyl moiety is available for glucosylation. Response surface methodology was applied to optimize the Gtf180-ΔN-Q1140E-catalyzed α-glucosylation of RebA, resulting in a highly productive process with a RebA conversion of 95% and a production of 115 g/L α-glucosylated products within 3 h. Development of a fed-batch reaction allowed further suppression of α-glucan synthesis which improved the product yield to 270 g/L. Sensory analysis by a trained panel revealed that glucosylated RebA products show a significant reduction in bitterness, resulting in a superior taste profile compared to RebA. The Gtf180-ΔN-Q1140E glucansucrase mutant enzyme thus is an efficient biocatalyst for generating α-glucosylated RebA variants with improved edulcorant/organoleptic properties.

## Introduction

**Figure 1:**
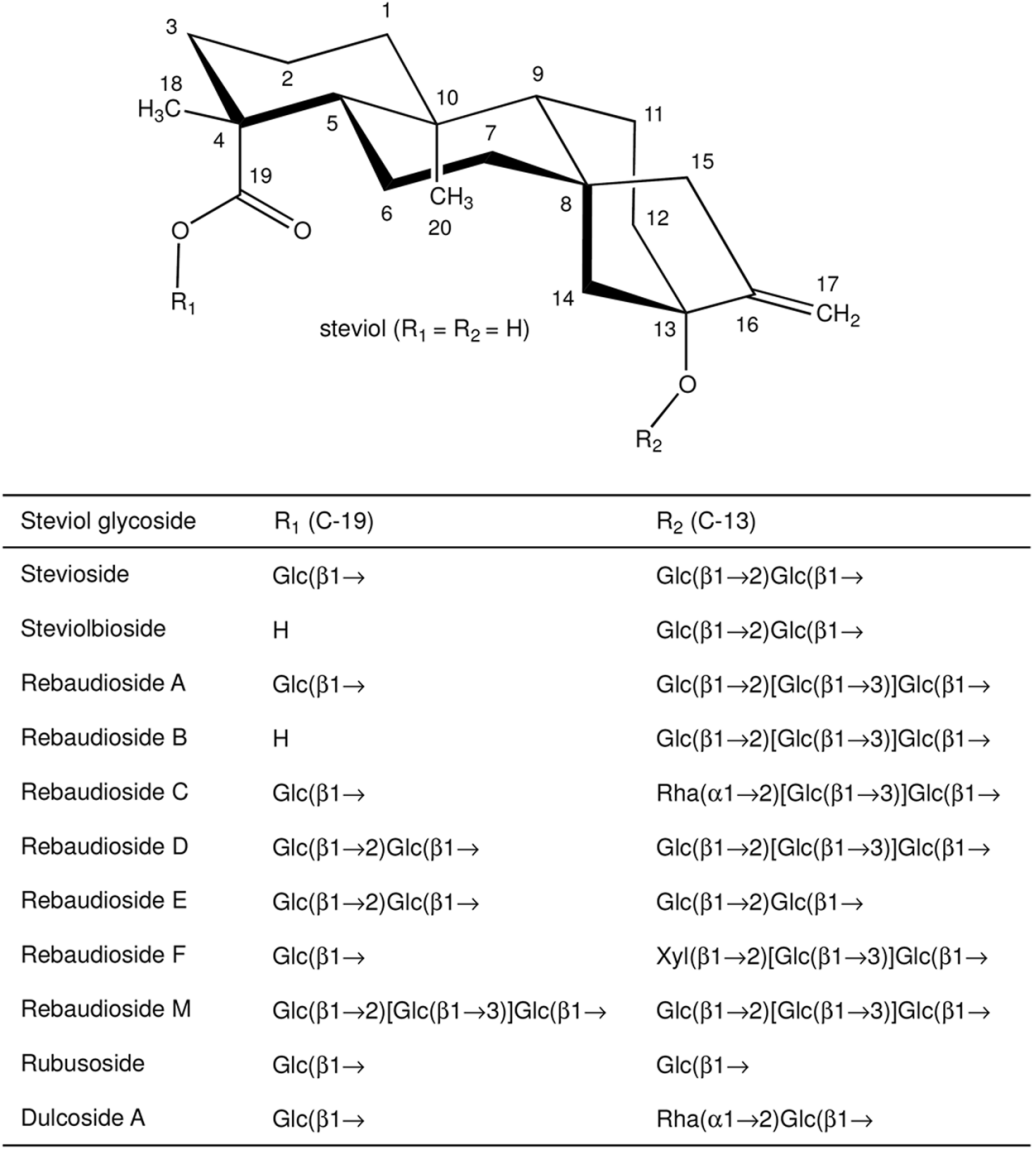
Structures of major steviol glycosides, occurring in the leaves of *Stevia rebaudiana*. Glucose (Glc), xylose (Xyl) and rhamnose (Rha) occur in the pyranose ring form. Glc and Xyl have D configuration and Rha L configuration.

The world-wide increasing incidence of obesity, diabetes type II, cardio vascular diseases, and dental caries leads to an increased consumer demand for food products and beverages without high-calorie sugars^1^. Steviol glycosides are excellent natural alternatives for sucrose and synthetic sweeteners^2–6^. These non-calorie compounds are extracted from the leaves of the herb plant *Stevia rebaudiana* BERTONI, a rhizomatous perennial shrub belonging to the Asteraceae [Compositae] family^7,8^. Stevioside (~5–20% w/w of dried leaves) and rebaudioside A (RebA) (~2–5% w/w of dried leaves) are the most abundant steviol glycosides, followed in lower concentrations by rebaudioside B, C, D, E, F, M, steviolbioside, rubusoside and dulcoside A (Fig. 1). Stevioside and RebA taste about 300 times sweeter than sucrose (0.4% aqueous solution). Steviol glycosides are approved as food additives in the USA since 2009 and they are on the European market (E 960, European Index) since December 2011^9^. Due to a genetic basis of taste perception, about half of the human population experiences a lingering bitter aftertaste with several steviol glycosides, the main drawback for their more successful commercialization as sweeteners^1,10^.

Structurally, steviol glycosides have *ent*-13-hydroxykaur-16-en-19-oic acid as aglycone, called steviol (Fig. 1)^8,11^. The presence and composition of the different carbohydrate moieties at the C-19-carboxylic acid group (R_1_) and at the C-13-*tert*-hydroxyl group (R_2_) of steviol determine the sweetness as well as the quality of taste of the steviol glycosides^12^. Sweetness increases and bitterness perception decreases with the total number of glycosyl residues^1^. The correlations bitterness/structure and sweetness/structure of steviol glycosides are not fully understood yet, particularly not in combination with the interactions with the human taste receptors^1,10,13,14^.

To improve the taste of steviol glycosides, especially for food applications, various (enzymatic) modifications of the carbohydrate moieties of steviol glycosides have been reported, mainly using cyclodextrin glycosyltransferase (CGTase), α- and β-glucosidase, α- and β-galactosidase and β-fructosidase transglycosylation and β-glycosyltransferase glycosylation systems as biocatalysts [see review^15^]. In the context of our study, the reports on trans-α-glucosylation are of interest. CGTases are attractive enzymes, which catalyze coupling and disproportionation reactions, transferring glucose residues from starch or cyclodextrins to acceptor molecules, yielding Glc(α1→4) extensions. Although often high yields are obtained with steviol glycosides, CGTases have poor steviol C-13/C-19 site regiospecificity, producing mixtures of steviol glycoside derivatives with mostly (α1→4)-glucosylation at both carbohydrate moieties^15,16^. Several early studies have shown that both mono- and di-(α1→4)-glucosylation of the carbohydrate moiety at the steviol C-13 site of stevioside and rubusoside gave products with a remarkable improvement in both intensity and quality of sweetness. However, (α1→4)-glucosylation of the Glc(β1→ residue at the steviol C-19 site resulted in an increased bitter aftertaste and a lower sweetness intensity^12,17–19^. α-Glucosylation of stevioside using Biozyme L (β-amylase preparation, probably contaminated with an α-glucosidase) and maltose as donor substrate, resulted in a product with a decreased sweetness, but a remarkable improvement in the quality of taste [Glc(α1→6) residue attached at the Glc(β1→C-19 residue], a product with a much lower sweetness [Glc(α1→6) residue attached to the terminal Glc(β1→2) residue of the β-sophorosyl-C-13 unit] and a product with a bitter taste [Glc(α1→3) residue attached to the terminal Glc(β1→2) residue of the β-sophorosyl-C-13 unit]^20^.

Aiming to obtain steviol glycoside derivatives with improved organoleptic properties, we studied the α-glucosylation potential of mutant glucansucrase enzymes of the generally recognized as safe (GRAS) bacterium *Lactobacillus reuteri* 180 on RebA. Glucansucrases (EC 2.1.4.5; glucosyltransferases, Gtfs) are extracellular enzymes catalyzing the synthesis of α-D-glucan polymers from the donor substrate sucrose, thereby introducing different ratios of glycosidic linkages [(α1→2), (α1→3), (α1→4), (α1→6)] in their glucan products, depending on the enzyme specificities^21,22^.

Recently, we have shown that the wild-type Gtf180-ΔN (N-terminally truncated) glucansucrase enzyme of *L*. *reuteri* 180 was able to glucosylate the steviol glycoside RebA, using sucrose as glucosyl donor substrate^23^. About 55% of RebA was glucosylated with up to eight α-D-glucosyl units attached (RebA-G). The formed RebA derivatives only had elongations at the steviol C-19 β-D-glucosyl moiety, mainly with alternating (α1→6)- and (α1→3)-linked glucopyranose residues, starting with an (α1→6) linkage (RebA-G1). In the present study, we have screened our in-house collection of Gtf180-ΔN glucansucrase mutant enzymes for variants with a better RebA glucosylating activity than the wild-type Gtf180-ΔN enzyme. One mutant was selected for more detailed studies and its biochemical characteristics and product profile were compared to wild-type Gtf180-ΔN. Glucosylated RebA products were isolated by flash chromatography and their structures were elucidated using MALDI-TOF mass spectrometry, methylation analysis and 1D/2D ^1^H/^13^C NMR spectroscopy. Furthermore, docking experiments with RebA and the available high-resolution 3D structure of Gtf180^24^ were carried out to evaluate the experimental data. Response surface methodology was applied to optimize the reaction conditions of RebA glucosylation with the selected mutant. Finally, sensory evaluations were performed to determine the taste attributes of the novel α-glucosylated RebA derivatives.

## Results

### Screening of wild-type Gtf180-ΔN and mutant glucansucrase enzymes for α-glucosylation of RebA

The RebA glucosylation activity of wild-type Gtf180-ΔN and 82 mutants derived was compared (Supplementary Tables S1 and S2). To this end, 50 mM RebA was incubated for 3 h with ~1 mg/mL of each enzyme in reaction buffer, containing either 0.2 M sucrose (for TLC product analysis) or 1 M sucrose (for HPLC product analysis). It has to be noted that the *in vivo* and most important activity of glucansucrases is the synthesis of α-D-glucan polymers and oligosaccharides from the donor substrate sucrose^22^. During the transglucosylation reaction with acceptor substrates such as steviol glycosides, the formation of α-gluco-oligo/polysaccharides is observed as a side-reaction, occurring to a varying extent depending on the specific (mutant) glucansucrase. The TLC (Supplementary Fig. S1) and HPLC (Supplementary Fig. S2) profiles obtained after the different enzyme incubations with RebA and sucrose clearly show that most of the Gtf180-ΔN mutants α-glucosylate RebA in similar amounts (based on spot intensity) as the wild-type Gtf180-ΔN enzyme (TLC lane 77). Mutant A978P (Supplementary Fig. S1, TLC lane 64) and mutants Q1140E and S1137Y (Supplementary Fig. S2), however, converted more RebA than the wild-type enzyme as can be seen by a smaller amount of residual RebA after the 3-h incubation. Some mutants, i.e. L981A (TLC lane 31), W1065L (TLC lane 71) and W1065Q (TLC lane 72), converted comparable amounts of RebA as the wild-type enzyme, but showed almost no polymerization activity, as indicated by the absence of the α-gluco-oligo/polysaccharide tails on TLC. Previously it has been shown that the mutations L981A, W1065L and W1065Q had a dramatic effect on the overall enzyme activity, resulting in a 93, 87 and 93% decrease at 100 mM sucrose, respectively^25,26^. Of all mutants tested, Q1140E showed the highest RebA conversion (Supplementary Fig. S2), and was therefore chosen for further studies, and compared to the wild-type Gtf180-ΔN enzyme. Mutant Gtf180-ΔN-Q1140E contains a single amino acid substitution (from a glutamine to a glutamate) close to the transition-state-stabilizing residue D1136^27^.

### Analytical details of α-glucosylated RebA products prepared with Gtf180-ΔN-Q1140E

For structural analysis purposes, a large-scale preparation of α-glucosylated RebA products was performed using 84 mM RebA, 282 mM sucrose and 5 U/mL Gtf180-ΔN-Q1140E enzyme (pH 4.7, 3 h, 37 °C). These incubation conditions were shown to be optimal in the Box-Behnken experimental design study, as described in the section “Optimization of the synthesis of α-glucosylated RebA” (see below). The used commercial RebA substrate is of high purity, as indicated by its ^1^H NMR spectrum (Supplementary Fig. S3; for the assignment of the resonances, see ref.^23^), MALDI-TOF mass spectrum (Supplementary Fig. S4), and methylation analysis (Supplementary Table S5). Contamination with other steviol glycosides was not detected, which is also of importance for the sensory analysis.

**Figure 2:**
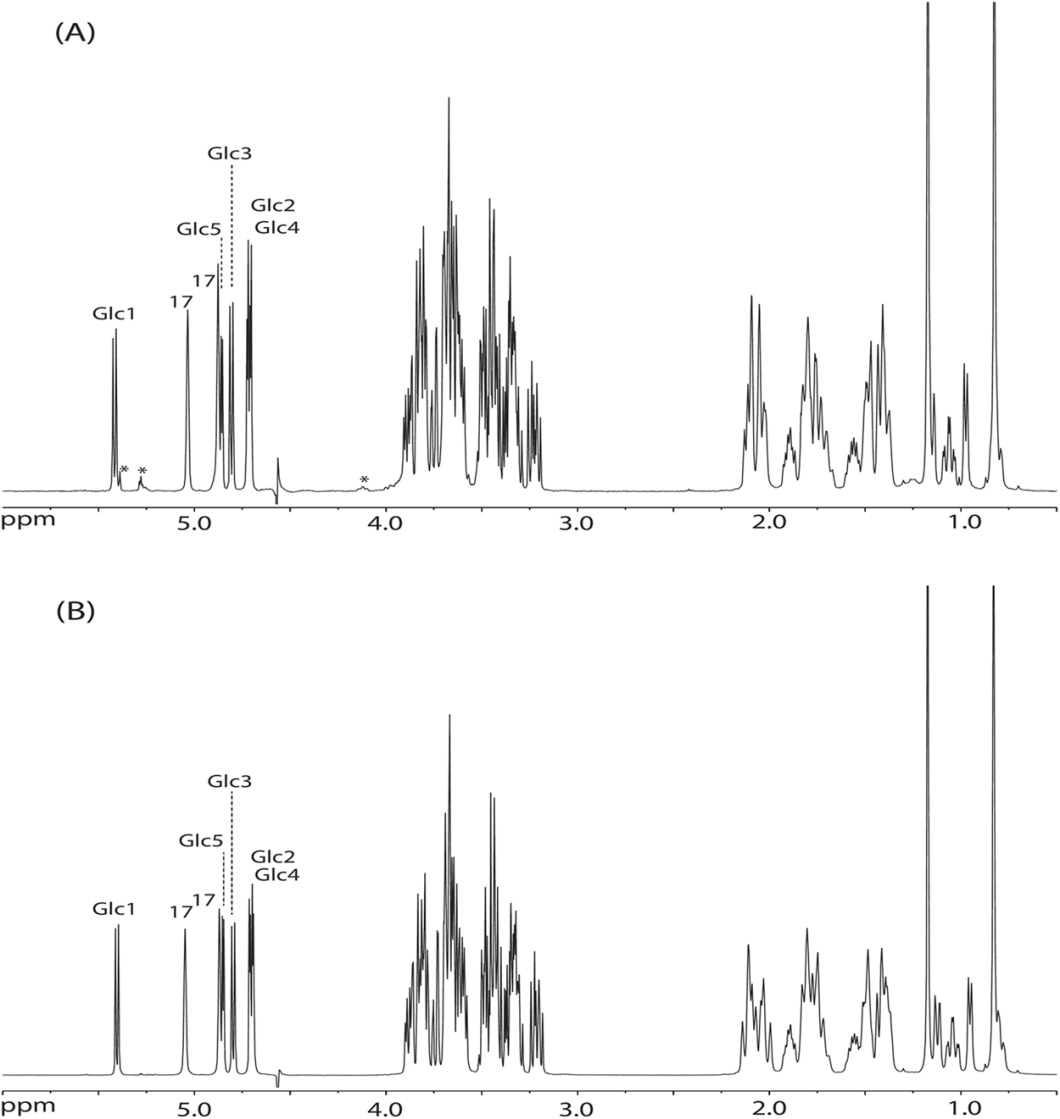
500-MHz ^1^H NMR spectra of **(A**) RebA-G and (**B**) RebA-G1, recorded in D_2_O at 310 K. The positions of the anomeric protons of the glucose residues (see Fig. 3) are indicated, as well as the steviol C-17 protons in the anomeric region. Products were synthesized using the mutant Gtf180-ΔN-Q1140E enzyme. Spectrum (**B**) is identical to that of RebA-G1, prepared with the wild-type Gtf180-ΔN enzyme, and recently published with a complete assignment of resonances^23^. *Signals stemming from higher α-glucosylated RebA products. Chemical shifts (δ) are expressed in ppm by reference to internal acetone (δ 2.225).

**Figure 3:**
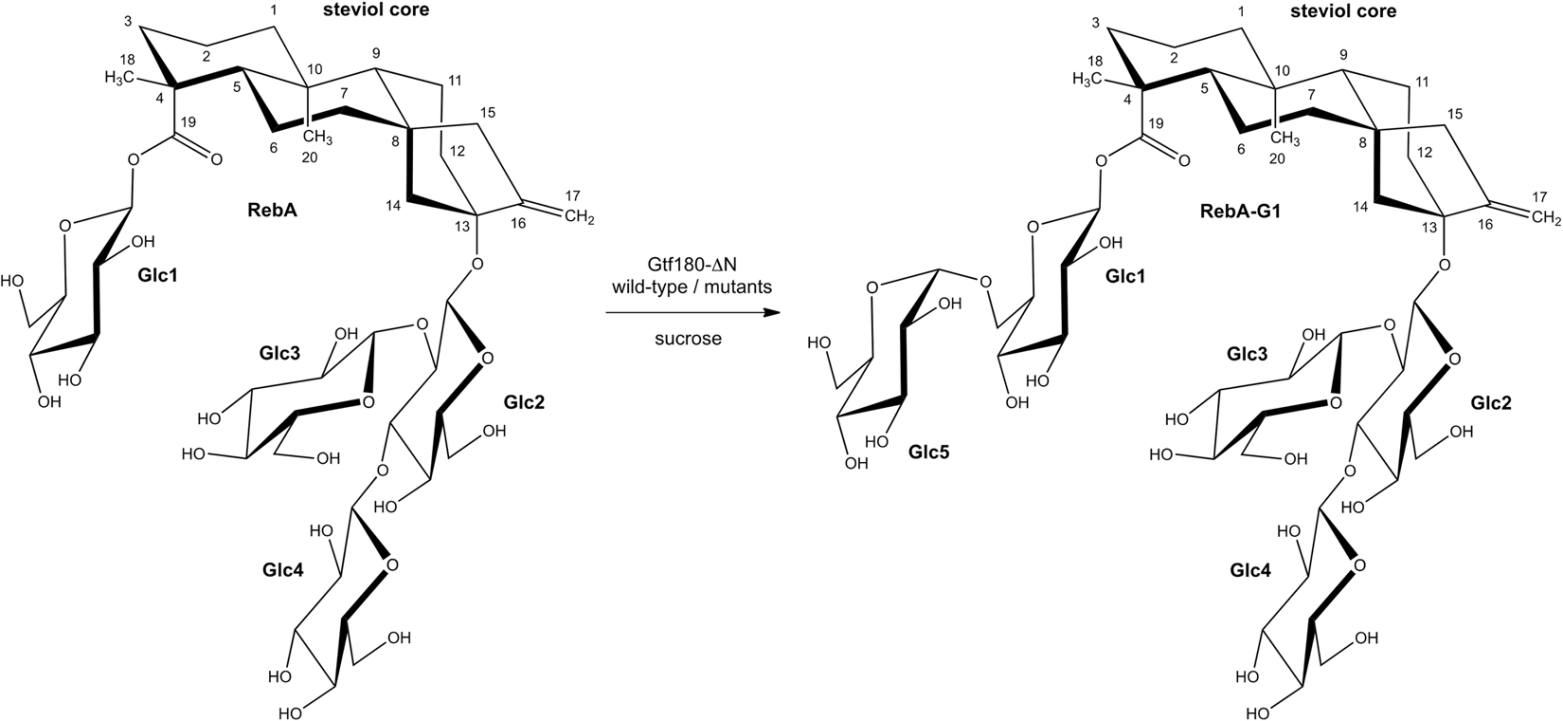
Major reaction product RebA-G1, obtained from the incubation of RebA with wild-type Gtf180-ΔN and mutant Gtf180-ΔN-Q1140E enzymes, in the presence of sucrose.

MALDI-TOF-MS analysis of the total RebA-incubation mixture (RebA-G) showed a series of quasi-molecular ions [M + Na]^+^, revealing remaining RebA (*m/z* 989.7) and extensions of RebA with one (major peak, *m/z* 1152.9) up to eight glucose residues (*m/z* 2287.9) (Supplementary Fig. S4). The ^1^H NMR spectrum of RebA-G (Fig. 2A) showed the typical steviol core signal pattern as seen for RebA (Supplementary Fig. S3). Besides the four β-anomeric ^1^H signals related to RebA (for structure, see Fig. 3; **Glc1**, δ 5.425; **Glc2**, δ 4.700; **Glc3**, δ 4.801; **Glc4**, δ 4.700), one additional α-anomeric ^1^H signal of similar intensity at δ 4.870 (**Glc5**) stemming from mono-α-glucosylated RebA was observed, together with very minor α-anomeric signals (marked with * in Fig. 2A) at δ 5.42 and δ 5.27, and a H-5 signal at δ 4.10, reflecting the presence of higher α-glucosylated RebA products (<10%). For full NMR details of these recently isolated higher α-glucosylated RebA products, see ref.^23^.

Flash chromatography fractionations were carried out to separate mono-α-glucosylated RebA (major fraction RebA-G1; MALDI-TOF-MS analysis: [M + Na]^+^, *m/z* 1152.9; Supplementary Fig. S4) from higher α-glucosylated RebA products (pooled very minor fractions RebA-G2 + ) and residual RebA. The ^1^H NMR spectrum of RebA-G1 (Fig. 2B) is identical to that reported recently for RebA-G1, prepared with the wild-type Gtf180-ΔN enzyme^23^. The corresponding 2D ^1^H-^13^C HSQC, TOCSY, and ROESY spectra of the carbohydrate part of RebA-G1 are depicted in Supplementary Fig. S5, and the ^1^H/^13^C chemical shifts in Supplementary Table S4. The very minor pool of higher α-glucosylated RebA fractions, RebA-G2+, was not used for further structural analysis. In summary, it can be concluded that mutant Gtf180-ΔN-Q1140E synthesizes as dominant product (77.7% in RebA-G) the same mono-α-glucosylated RebA derivative RebA-G1 as the wild-type Gtf180-ΔN, i.e. a product with a specific elongation of the steviol C-19 β-D-Glc*p* moiety of RebA with an α-D-Glc*p*-(1→6) residue (Fig. 3). Taking into account the structural data of the α-glucosylated RebA products isolated in the wild-type Gtf180-ΔN/RebA/sucrose incubation study^23^, combined with the above-mentioned extra minor signals in the ^1^H NMR spectrum of RebA-G (Fig. 2A), it can be concluded that also in the case of the Gtf180-ΔN-Q1140E/RebA/sucrose incubation, RebA-G1 is further extended at the C-19 site with mainly alternating (α1→3)- and (α1→6)-linked D-Glc*p* residues. The methylation analysis data of RebA-G and RebA-G1, presented in Supplementary Table S5, support the NMR data.

### Optimization of the synthesis of α-glucosylated RebA

#### Batch reaction

RebA is only sparingly soluble in water (<10 mM) at room temperature, however, it readily forms supersaturated solutions in water on simple stirring^28^. It was observed that up to 200 mM RebA could be dissolved at 37 °C before it started precipitating at 90 min. Hence, an efficient conversion of 200 mM RebA into glucosylated products has to be achieved within 90 min in order to prevent a suboptimal glucosylation yield caused by precipitation of RebA. Important factors for an optimal conversion of RebA into α-glucosylated RebA (RebA-G) are the RebA concentration, the ratio of donor substrate sucrose over acceptor substrate RebA (D/A ratio) and the agitation speed.

**Figure 4:**
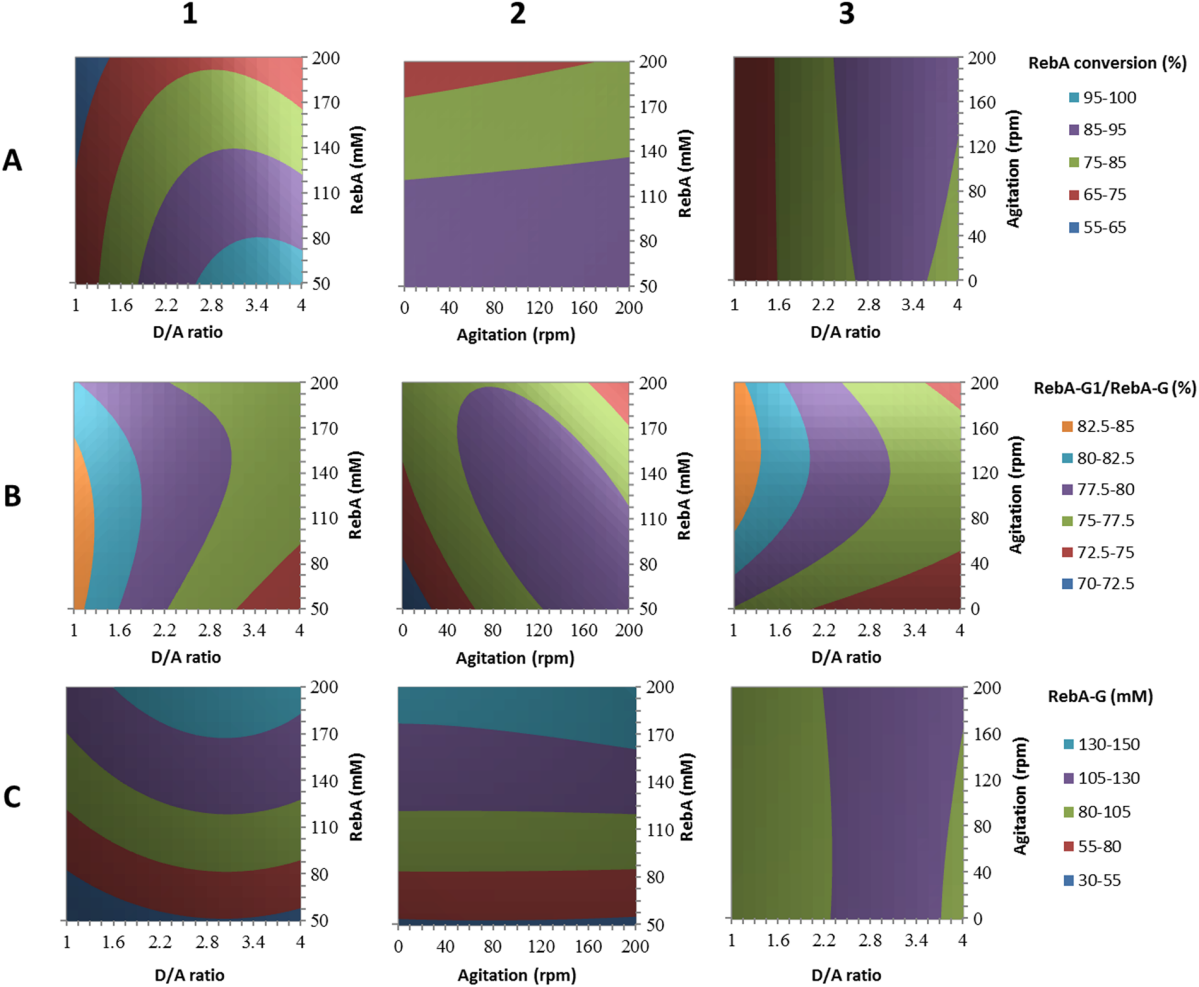
Response surface methodology contour plots of RebA glucosylation by Gtf180-ΔN-Q1140E, showing the effects of RebA concentration (mM), D/A ratio (ratio of donor substrate sucrose over acceptor substrate RebA) and agitation (rpm) on **(A)** RebA conversion (%); **(B)** RebA-G1/RebA-G ratio (%); **(C)** RebA-G synthesized (mM).

A response surface methodology (RSM) using a Box-Behnken experimental design was performed considering three factors: X_1_, RebA concentration (mM); X_2_, D/A ratio; X_3_, agitation speed (rpm). The addition of 5 U/mL Gtf180-ΔN-Q1140E enzyme ensured that a steady state in RebA conversion was obtained well before precipitation could occur for the highest RebA concentration (90 min). The results of the Box-Behnken experimental design are summarized in Supplementary Table S3. The analysis of variance (ANOVA) showed R^2^ values of 98.8%, 78.0% and 99.3% for RebA conversion degrees (%), RebA-G1/RebA-G ratio (%) and amount of RebA-G synthesized (mM), respectively. The effects of the factors were analyzed applying the response surface contour plots (Fig. 4).

In summary, RebA conversion degrees decreased with increasing RebA concentrations, independent of the sucrose concentration (Fig. 4A1). The RebA conversion degrees displayed an optimum at a D/A ratio of 2.5–3.5, depending on the RebA concentration (Fig. 4A1–A3). Increasing the D/A ratio initially resulted in improved RebA conversion degrees, reflecting that more sucrose was available to drive the reaction. A further increase of the D/A ratio resulted in less RebA glucosylation in favour of more α-glucan synthesis. Agitation had only a slight effect on RebA conversion degrees and amount of RebA-G synthesized (Fig. 4A2–C2). Agitation influenced the RebA-G1/RebA-G ratio more strongly: the highest ratios were observed at an agitation speed of approximately 185 rpm (Fig. 4B2–B3). Furthermore, low D/A ratios favoured high RebA-G1/RebA-G ratios, since less donor substrate sucrose was available to glucosylate RebA-G1 into multi-glucosylated products (Fig. 4B1).

**Figure 5:**
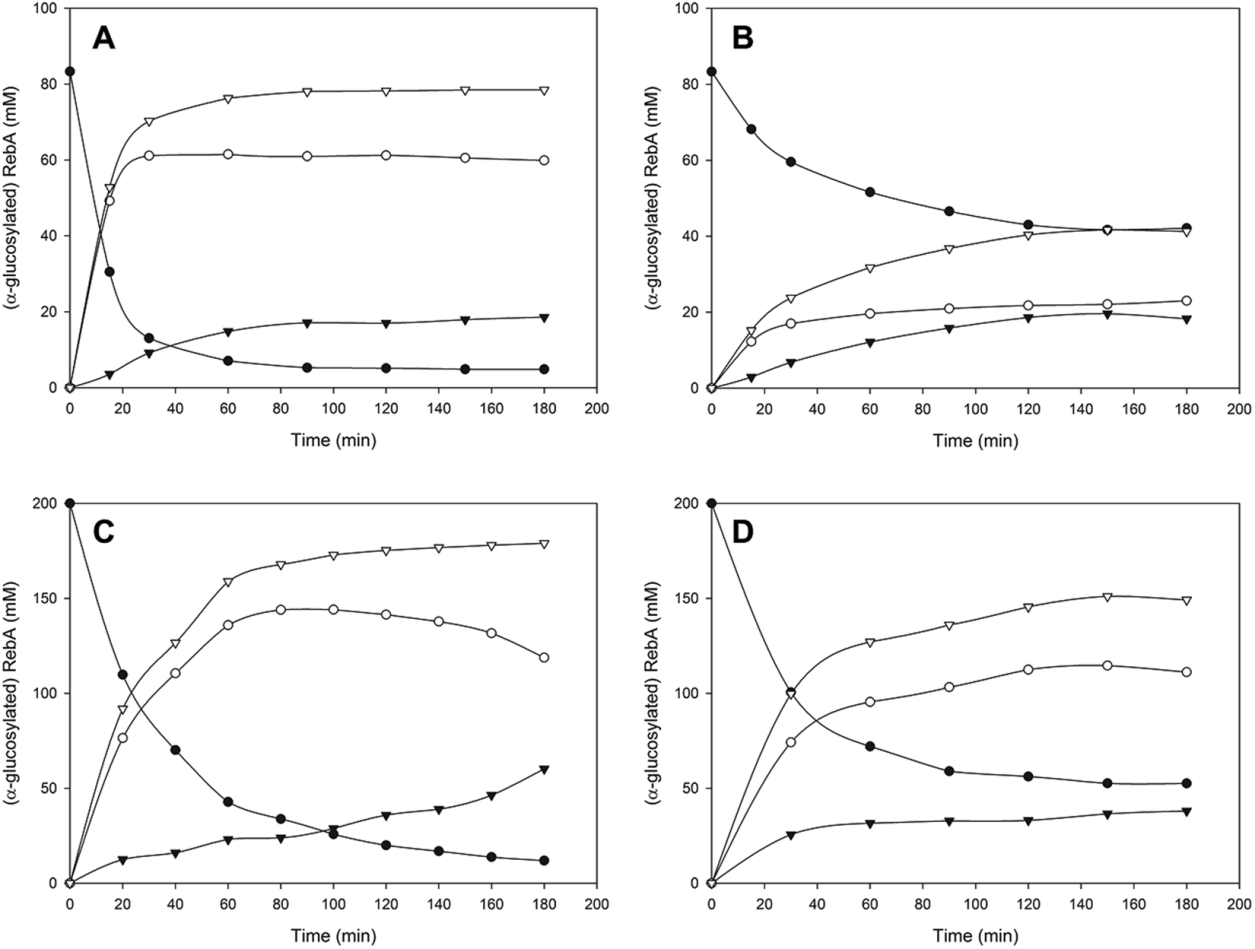
Time course of RebA glucosylation by **(A)** Gtf180-ΔN-Q1140E at optimal batch conditions (84 mM RebA; 282 mM sucrose; 5 U/mL), **(B)** Gtf180-ΔN at optimal batch conditions (84 mM RebA; 282 mM sucrose; 5 U/mL), **(C)** Gtf180-ΔN-Q1140E at optimal fed-batch conditions (200 mM RebA; 50 mM sucrose; 50 U/mL) and **(D)** Gtf180-ΔN-Q1140E at batch conditions (200 mM RebA; 570 mM sucrose; 5 U/mL). • RebA, ○ RebA-G1, ▾ RebA-G2+, ▽ RebA-G. For the definition of these products, see Methods “Design of response surface methodology experiment”.

The resulting Box-Behnken model (Supplementary Table S3) was subsequently used for the optimization of the reaction conditions. An efficient conversion of RebA into RebA-G yielding a maximal amount of RebA-G (at least 95%) was aimed for. The model predicted the synthesis of 80 mM RebA-G for the following conditions: 5 U/mL Gtf180-ΔN-Q1140E, 84 mM RebA, 282 mM sucrose and 185 rpm. The validation test resulted in the synthesis of 79 mM (or 115 g/L) RebA-G with a RebA-G1/RebA-G ratio of 77.7% (Fig. 5A), which was in very good agreement with the prediction. Applying identical conditions for the glucosylation of RebA with wild-type Gtf180-ΔN resulted only in conversion of 49.7% RebA, yielding 42 mM RebA-G with a RebA-G1/RebA-G ratio of 54.4% (Fig. 5B). Hence, the Q1140E-mutant not only converted more RebA into RebA-G than wild-type Gtf180-ΔN (94.5% vs. 49.7%), its RebA-G consisted mostly of mono-α-glucosylated product RebA-G1 (77.7% vs. 54.4%). Compared to wild-type (45.6%), Q1140E produced less RebA-G2+ (22.3%), which is the minor fraction of the RebA glucosides with 2 and more glucose units.

#### Fed-batch reaction

The main remaining bottleneck for RebA glucosylation with Gtf180-ΔN-Q1140E is the synthesis of α-glucans at high sucrose concentrations, preventing complete RebA glucosylation at high RebA concentrations (Fig. 4A1). This issue was addressed by performing a fed-batch reaction, in which sucrose was added to the reaction in fixed intervals of 20 min in order to keep the sucrose concentration low and hence suppress α-glucan formation as much as possible. The addition of 50 U/mL enzyme ensured complete usage of sucrose within 20 min and complete conversion of RebA within 3 h. Figure 5C represents RebA glucosylation at 200 mM RebA and an average sucrose concentration of 50 mM (fluctuating between 0–100 mM). In comparison to the batch reaction (200 mM RebA, 570 mM sucrose) (Fig. 5D), the RebA fed-batch conversion (Fig. 5C) increased from 76.4% to 94.1%, attributed to a further suppressed α-glucan synthesis. The product yield consequently increased to 188 mM (or 270 g/L) RebA-G (Fig. 5C).

### Docking of RebA in the active site of wild-type Gtf180-ΔN and mutant Q1140E

To gain further insight into the α-glucosylation of RebA by the Gtf180-ΔN wild-type enzyme and its mutant Gtf180-ΔN-Q1140E, *in silico* docking studies^29^ were performed using the crystal structure of RebA^30^.

**Figure 6:**
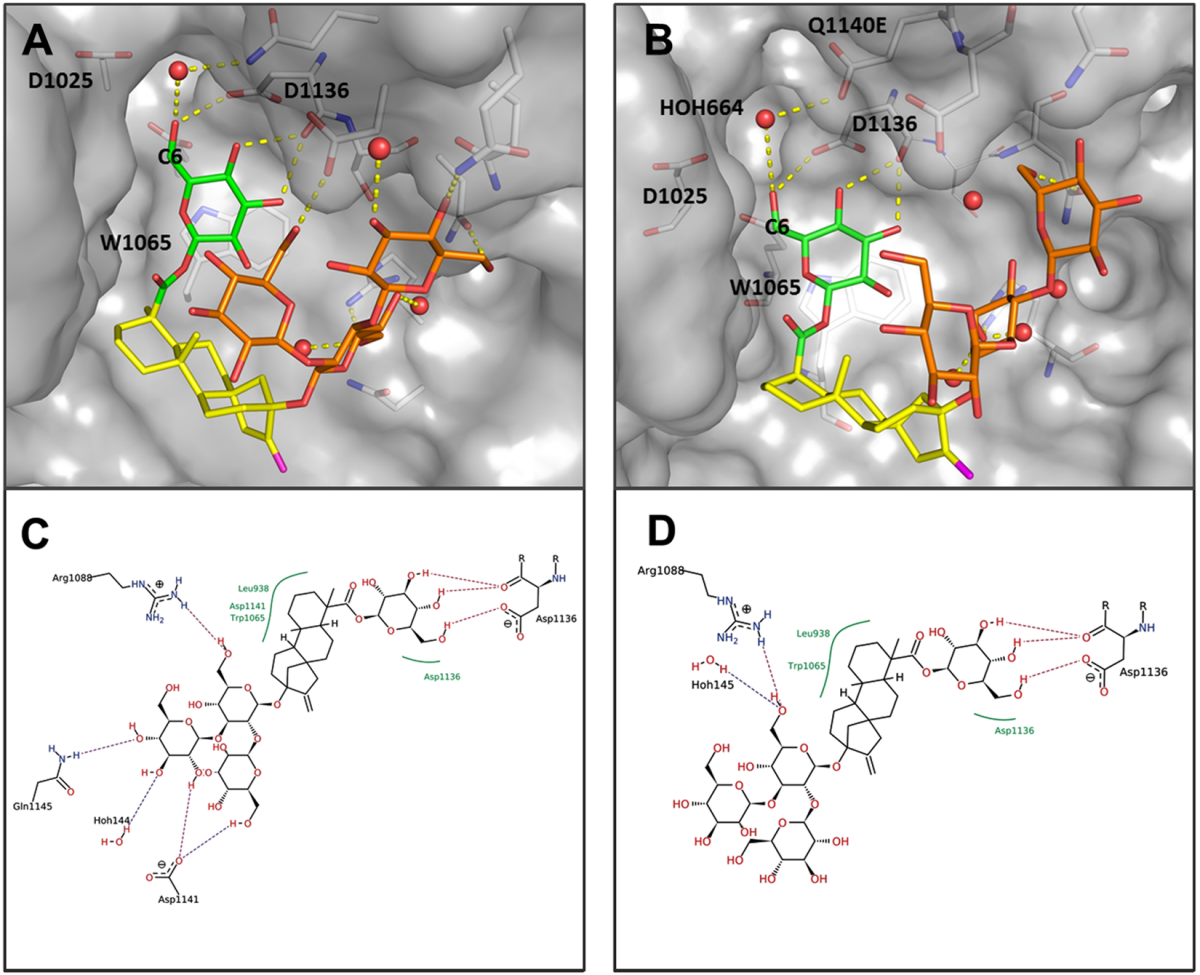
Docking poses of RebA into wild-type Gtf180-ΔN (PDB: 3KLL; **A** and **C**) and mutant Q1140E (**B** and **D**). The steviol part is indicated in yellow, the **Glc1**(β1→ residue at the steviol C-19 site in green and the **Glc3**(β1→2)[**Glc4**(β1→3)]**Glc2**(β1→ trisaccharide at the steviol C-13 site in orange. The monosaccharide coding system used here is as shown in Fig. 3.

Docking of RebA into the wild-type Gtf180-ΔN active site (X-ray crystal structure of a complex of Gtf180-ΔN with maltose; PDB code: 3KLL^24^) afforded the pose as depicted in Fig. 6A, showing that only the steviol C-19 β-D-glucosyl moiety (**Glc1** in Fig. 3) of RebA is available for glucosylation, especially at the HO-6 group, due to its orientation. Hydrogen bonding of **Glc1** HO-6 with the catalytic residue D1136 possibly supports deprotonation in the glucosylation step (Fig. 6C). A π–π-stacking interaction with W1065 as well as hydrogen bonding of **Glc1** HO-4 and HO-3 to the backbone of D1136 was observed and appears to hold the **Glc1** residue in place.

Docking of RebA into the active site of the mutant Gtf180-ΔN-Q1140E afforded the pose as depicted in Fig. 6B. The binding of the steviol C-19 β-D-glucosyl moiety (**Glc1** in Fig. 3) is identical to the wild-type enzyme, but fewer hydrogen bonds of the steviol C-13 trisaccharide moiety (**Glc2**, **Glc3** and **Glc4** in Fig. 3) were observed (Fig. 6D). The modified binding pocket, resulting from the mutation, does not influence the binding of the steviol C-19 β-D-glucosyl unit, given that the mutation is not in direct proximity of the monosaccharide. The experimental observation that mutant Q1140E leads to more efficient α-glucosylation could be explained by the possibility that deprotonation of **Glc1** HO-6 is aided by a water-mediated hydrogen bond between **Glc1** HO-6 and E1140 as depicted in Fig. 6B. The finding that the Q1140E mutant shows mostly mono-glucosylation instead of oligo-glucosylation is currently difficult to explain on the basis of these docking results.

### Sensory analysis of glucosylated RebA products

**Figure 7:**
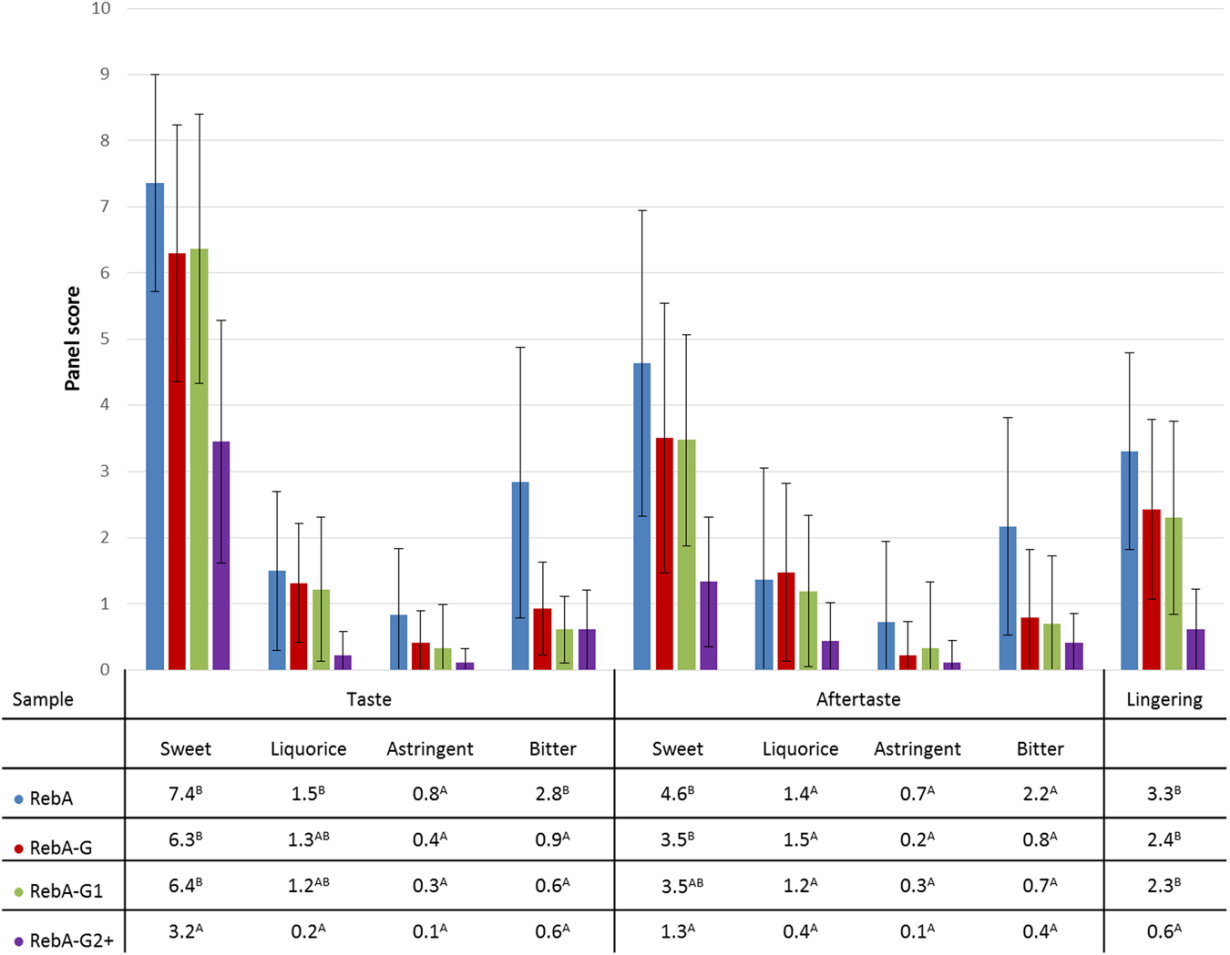
Sensory analysis of RebA, RebA-G, RebA-G1 and RebA-G2+. ^A,B^Different letters indicate significant differences (p < 0.05) between solutions following one-way ANOVA and post-hoc test.

A sensory analysis of aqueous solutions sweetened with RebA and glucosylated RebA products, prepared with the mutant Gtf180-ΔN-Q1140E enzyme, was performed by a trained panel, evaluating 9 different taste attributes. Three different glucosylated products were examined: multi-α-glucosylated product, containing residual RebA (RebA-G), mono-α-glucosylated product (RebA-G1) and RebA-G lacking RebA and RebA-G1 (RebA-G2+). The mean scores of the attributes of the sweetened water solutions are shown in Fig. 7.

The glucosylated RebA products were all significantly less bitter than RebA, in fact they displayed almost no bitterness at all. Equally important, RebA-G and RebA-G1 retained the very high sweetness inherent to RebA. In contrast, RebA-G2+ was significantly less liquorice, astringent and lingering than RebA but also significantly less sweet, retaining only half of the RebA sweetness. The sensory analysis also revealed that RebA-G and RebA-G1 have very similar taste profiles, both combining a very high sweetness with a very low bitterness and other off-flavours. So, the small amounts of RebA and RebA-G2+ in RebA-G apparently do not influence the taste profile, when compared with RebA-G1. Therefore, RebA-G is the preferred product for commercialization, since it can be produced more economically, not requiring further purification and separation steps.

## Discussion

The *Stevia rebaudiana* plant is a major source of high-potency natural sweeteners (steviol glycosides) for the growing natural food market of the future^3^, however, due to their slight bitterness and unpleasant and lingering aftertaste, large-scale application of steviol glycosides is still hampered. In the past, several attempts have been made to improve the quality and sweet taste of steviol glycosides by modifying the carbohydrate moieties at the C-13 *tert*-hydroxyl and the C-19 carboxylic acid functions of steviol via transglycosylation reactions [for a recent review, see ref.^15^].

In the present study, we have successfully used the glucansucrase mutant enzyme Gtf180-ΔN-Q1140E from *Lactobacillus reuteri* 180 (with sucrose as donor substrate) to glucosylate the steviol glycoside RebA, an important *Stevia* component. In a screening of 82 mutants of Gtf180-ΔN, mutant Q1140E was selected for further studies and compared to wild-type Gtf180-ΔN. At optimal conditions, mutant Q1140E achieved ~95% RebA conversion into mainly mono-α-glucosylated RebA product RebA-G1 (Fig. 3), compared to only 55% conversion by the wild-type enzyme. Under batch conditions, a high product yield of 115 g/L RebA-G (79 mM) was obtained within 3 h (from 84 mM RebA), applying only 5 U/mL of the Q1140E mutant enzyme. The product yield could even be enhanced to 270 g/L RebA-G (188 mM) (from 200 mM RebA) by adopting a fed-batch reaction with stepwise addition of sucrose. This reduced availability of sucrose effectively suppressed the formation of α-gluco-oligo/polysaccharides. Instead, sucrose was mainly used as donor substrate for RebA glucosylation by the Q1140E enzyme, yielding more RebA α-glucosides.

The Gtf180-ΔN-Q1140E mutant glucosylated RebA specifically at the steviol C-19 position, introducing a Glc(α1→6) residue at the ester-linked Glc(β1→ residue. This finding is in line with the present knowledge about the wild-type Gtf180-ΔN enzyme, which also specifically elongates the C-19 glucose residue with mainly alternating (α1→6)- and (α1→3)-linked glucose units^23^. In contrast to the modified steviol glycosides prepared by incubation with CGTases, thereby introducing only (α1→4)-linked glucose residues, the (α1→3) and (α1→6) linkages are resistant to hydrolysis by the human amylolytic enzymes in saliva, which may prolong the sweet taste in the mouth.

Molecular docking studies were performed to gain insight into the glucosylation mechanism of RebA at the molecular level and to elucidate how a single amino acid change in Gtf180-ΔN, namely Q1140E, significantly improved RebA conversion. Docking of RebA into the active site of the Gtf180-ΔN wild-type enzyme indicated that only its steviol C-19-ester-linked Glc(β1→ residue is available for glucosylation. The Q1140E mutation is predicted not to affect the orientation and position of RebA in the active site, supporting the experimental observation that both enzymes specifically α-glucosylate RebA at the **Glc1**(β1→C-19 residue, and not at the **Glc3**(β1→2)[**Glc4**(β1→3)]**Glc2**(β1→C-13 trisaccharide (Fig. 3). Furthermore, the docking results showed that **Glc1** HO-6 and not **Glc1** HO-3 of the **Glc1**(β1→C-19 residue of RebA is prominently available for glucosylation. This is in agreement with the experimental results that both wild-type and mutant Gtf180-ΔN enzymes attach the first Glc residue (**Glc5**) exclusively via an (α1→6)-linkage. A faster and more efficient glucosylation of RebA was obtained by replacement of glutamine with the more negatively charged glutamate at position 1140 (mutant Q1140E). Conceivably, deprotonation of **Glc1** HO-6 is aided by a water-mediated hydrogen bond between **Glc1** HO-6 and glutamate, which is absent with glutamine at position 1140 (Fig. 6). The finding that Q1140E shows mostly mono-glucosylation is currently difficult to explain on basis of the docking results. Elucidation of the Q1140E mutant protein 3D structure, followed by a comparison of the Gtf180-ΔN wild-type and mutant Q1140E structures, ideally in complex with RebA, may shed more light on the observed differences in RebA glucosylation.

An important finding in our study was that the multi-glucosylated RebA product, RebA-G, had a significantly reduced bitterness compared to RebA. This improved steviol glycoside product mixture thus displays appealing sensory properties and is likely to find application as a functional food ingredient. This study also shows that Gtf180-ΔN-Q1140E is a very efficient catalyst for α-glucosylation of steviol glycosides.

## Methods

### Glucansucrase enzymes

Gtf180-ΔN is the 117-kDa N-terminally truncated (741 residues) fragment of the wild-type Gtf180 full-length protein, derived from *L*. *reuteri* strain 180^31^. In Gtf180-ΔN-ΔV both the N-terminal variable domain and the N-terminal domain V fragment (corresponding to the first 793 N-terminal amino acids), and the C-terminal domain V fragment (corresponding to the last 136 C-terminal amino acids) have been deleted^32^. The glucansucrase Gtf180-ΔN mutant enzymes were constructed using QuikChange site-directed mutagenesis (Stratagene, La Jolla, CA)^25–27,31–35^ (see Supplementary Table S1).

### Standard reaction buffer

All enzymatic reactions were performed at 37 °C in 25 mM sodium acetate (pH 4.7), containing 1 mM CaCl_2_.

### Enzyme-activity assays

Enzyme activity assays were performed at 37 °C in reaction buffer with 100 mM sucrose. Samples of 100 μL were taken every min over a period of 7 min and immediately inactivated with 20 µL 1 M NaOH. The glucose and fructose concentrations were enzymatically determined by monitoring the reduction of NADP with the hexokinase and glucose-6-phosphate dehydrogenase/phosphoglucose isomerase assays (Roche Nederland BV, Woerden, The Netherlands)^36^. Determination of the release of glucose and fructose from sucrose allowed calculation of the total activity of the glucansucrase enzymes^37^. One unit (U) of enzyme is defined as the amount of enzyme required for producing 1 μmol fructose per min in reaction buffer, containing 100 mM sucrose at 37 °C.

### Screening of (mutant) glucansucrases for enzymatic α-glucosylation of RebA

In an initial screening, six Gtf180-ΔN-derived mutants were compared to wild-type Gtf180-ΔN by analyzing reaction products with high-performance liquid chromatography (HPLC). Then, an additional 76 Gtf180-ΔN mutants from our collection (Supplementary Table S2) were screened and analyzed using thin-layer chromatography (TLC). Incubations of 3 h were performed in reaction buffer, containing ~1 mg/mL enzyme, 50 mM RebA (Sigma-Aldrich Chemie, Zwijndrecht, The Netherlands) and 1 M sucrose (for HPLC analysis) or 0.2 M sucrose (for TLC analysis). For HPLC analysis, 10 µL of the incubation mixture was diluted in 250 µL 80% methanol and centrifuged for 2 min at 15,000 × *g*. Then, 40 µL of the upper phase was injected on a Luna 10 μm NH_2_ column (250 × 4.6 mm; Phenomenex, Utrecht, The Netherlands). Separation was obtained at a flow-rate of 1 mL/min under gradient elution conditions (solvent A = acetonitrile; solvent B = 0.025% acetic acid in H_2_O), starting with a 2-min isocratic step of 70% solvent A followed by a linear gradient from 70 to 55% solvent A over 9 min and a final 3-min washing step of 20% solvent A. HPLC analyses were performed using an UltiMate 3000 chromatography system, equipped with a VWD-3000 UV-vis detector (ThermoFisher Scientific, Amsterdam, The Netherlands; monitoring at 210 nm). For TLC analysis, 1 µL of the enzymatic reaction mixtures was spotted on TLC sheets (Kieselgel 60 F254, 20 × 20 cm; Merck, Darmstadt, Germany), which were developed in *n*-butanol/acetic acid/water (2:1:1, v/v/v). After drying of the sheets, the bands were visualized by orcinol/sulfuric acid staining.

### Quantitative preparation of α-glucosylated RebA products

Incubations of 84 mM RebA (Tereos PureCircle Solutions, Lille, France; 97% purity, HPLC grade) were performed in 50 mL reaction buffer with 282 mM sucrose donor substrate, using 5 U/mL Gtf180-ΔN-Q1140E enzyme, during 3 h. Fractionations of RebA-G were carried out by flash chromatography using a Reveleris X2 flash chromatography system (Büchi Labortechnik AG, Flawil, Switzerland) with a Reveleris C18 cartridge (12 g, 40 µm) with water (solvent A) and acetonitrile (solvent B) as the mobile phase (30 mL/min). The following gradient elution was used: 95% solvent A (0–2 min), 95–50% solvent A (2–20 min), 50–95% solvent B (20–22 min), 95% solvent B (22–25 min). The collected fractions were evaporated *in vacuo* and subsequently freeze dried to remove the residual water.

### Design of response surface methodology experiment

Response surface methodology^38^ was applied to optimize the glucosylation of RebA. A Box-Behnken design^39^ was generated implementing RebA concentration (mM), sucrose/RebA ratio and agitation rate (rpm) as factors. For each of them low (−1) and high (+1) level values were assigned as follows: RebA concentration (50 mM) and (200 mM), sucrose/RebA ratio (1:1) and (4:1), agitation rate (0 rpm) and (200 rpm). The addition of 5 U/mL Gtf180-ΔN-Q1140E enzyme ensured a steady-state was reached within 3 h of incubation. The experimental design was generated and analyzed using JMP software (release 12)^40^ and consisted of 15 experiments carried out at 50-mL scale in shake flasks, continuously mixed by shaking (Supplementary Table S3). Results were analyzed with HPLC (see below). The response surface analysis module of JMP software was applied to fit the following second order polynomial equation (1):

1 $$\hat{Y}={\beta }_{0}+\sum _{i=1}^{I}{\beta }_{i}{X}_{i}+\sum _{i=1}^{I}{\beta }_{ii}{X}_{i}^{2}+\,\sum _{i}\sum _{j}^{\,}{\beta }_{ij}{X}_{i}{X}_{j}$$

where $\hat{Y}$ is the predicted response, $I$ is the number of factors (3 in this study), ${\beta }_{0}$ is the model constant, ${\beta }_{i}$ is the linear coefficient associated to factor ${X}_{i}$, ${\beta }_{{ii}}$ is the quadratic coefficient associated to factor ${X}_{i}^{2}$ and ${\beta }_{{ij}}$ is the interaction coefficient between factors ${X}_{i}$ and ${X}_{j}$. ${X}_{i}$ represents the factor variable in coded form (equation 2):

2 $${X}_{c,i}=\frac{[{X}_{i}-(low+high)/2]}{(high-low)/2}$$

With 1 ≤ *i* ≤ *l*, where ${X}_{c,i}$ is the coded variable.

For the HPLC analysis of the RebA and glucosylated RebA products, an Agilent ZORBAX Eclipse Plus C18 column (100 × 4.6 mm, 3.5 µm) was used with water (solvent A) and acetonitrile (solvent B) as the mobile phase. The flow rate and temperature were set at 1.0 mL/min and 40 °C, respectively. The following gradient elution was used: 5–95% solvent B (0–25 min), 95% solvent B (25–27 min), 95–5% solvent B (27–30 min) and again 95% of solvent A (30–35 min). Detection was achieved with an ELSD detector (evaporation temperature, 90 °C; nebulization temperature, 70 °C; gas flow rate, 1.6 SLM). Calibration of the obtained peaks for RebA and mono-glucosylated RebA (RebA-G1) was accomplished using the corresponding standard curves. In this context, the concentration of multi-glucosylated product (RebA-G) at a specific time was calculated as the initial RebA concentration minus the RebA concentration at that time. Multi-glucosylated RebA product lacking RebA-G1 (defined as RebA-G2+) was equal to RebA-G minus RebA-G1.

### Methylation analysis, mass spectrometry and NMR spectroscopy

For details of methylation analysis, gas-liquid chromatography – electron impact mass spectrometry (GLC-EIMS), matrix-assisted laser-desorption ionization time-of-flight mass spectrometry (MALDI-TOF-MS), and 1D/2D/(^1^H, TOCSY, ROESY, HSQC) NMR spectroscopy, see ref.^23^.

### Molecular docking of RebA in the active site of Gtf180-ΔN and Gtf180-ΔN-Q1140E

The X-ray crystal structure of Gtf180-ΔN complexed with maltose (PDB code: 3KLL^24^) was used for docking by using LeadIT 2.1.8 from BiosolveIT^29^. The acceptor binding site was defined by manual selection and included the following amino acid residues: 935–941, 944, 964–968, 975–983, 985, 1023–1032, 1035, 1061–1069, 1082–1093, 1096, 1111, 1129–1142, 1144, 1145, 1155, 1183, 1202, 1204, 1407, 1409, 1411, 1412, 1443, 1446, 1456–1458, 1463–1466, 1504–1509, 1511, 1526, 1527, and the water molecules numbered 7, 15, 45, 83, 106, 144, 145, 172, 401, 432–436, 466, 469, 470, 527, 572, 605, 648, 666, which have at least two interactions. The crystal structure of rebaudioside A.4H_2_O.1CH_3_OH was taken from the literature (CSD entry: DAWCEL)^30^, and the water and methanol were removed. The default settings were used for docking, except for the following features: the docking strategy was chosen to be driven by Entropy (Single Interaction Scan), a hard enzyme was used (maximum allowed overlap volume: 2 Å) and the maximum number of solutions per fragmentation was increased to 400. In total, 30 poses were generated with LeadIT, using the scoring function HYDE in SeeSAR^41^, ranked according to their estimated affinity; finally, poses with torsions flagged in red were removed. Visual inspection of the residual poses and deletion of unreliable poses resulted in a trustable set of poses. The model of mutant Q1140E, which was used for the docking experiment, was built in PyMOL^42^ and the rotamer showing the smallest number of steric clashes was chosen.

### Sensory analysis

Panellists were selected on basis of their performance on basic taste recognitions, ability to ascertain degrees of differences for specific taste stimuli at different concentrations and repeatability^43^, as verified by triangle tests and Wald sequential analysis^44^. Following the selection, the panel was trained over a 6-month period. In a first session, the panellists had to taste RebA solution at 10% (w/w) sucrose equivalent level. Their own vocabulary was used to describe taste and off-tastes as well as aftertastes. In the second session, the following attributes were selected from the first session and from literature^45–47^: sweetness, liquorice, astringency and bitterness. In the following months, training sessions were alternated with discussion sessions to agree on scoring of sweetness, off-tastes, aftertastes and lingering on a 15 point hedonic scale, and evaluation protocol.

The actual Quantitative Descriptive Analysis (QDA) was performed in individual tasting booths at the UGent Sensolab (Belgium) by the trained panel (9 persons). The fixed evaluation protocol with standardized vocabulary was applied. Firstly, taste (sweetness, liquorice, astringency and bitterness) was evaluated by swirling the sample in the mouth for 5 sec after which the sample was expectorated. Secondly, aftertaste was evaluated 10 sec after swallowing the solution. Next, lingering based on the maximum taste intensity was rated 1 min later. Sucrose reference solutions (5%, 7.5% and 10% sucrose scoring 5, 7.5 and 10, respectively) were provided. Water (Spa Reine**)** and plain crackers were used as palate cleansers between sampling. All samples were evaluated in duplicate.

Statistical analyses were performed with SPSS 23 (SPSS Inc., Chicago, USA). All tests were done at a significance level of 0.05. One-Way ANOVA was used to investigate any significant difference between the solutions. Testing for equal variances was executed with the Modified Levene Test. When conditions for equal variance were fulfilled, the Tukey test^48^ was used to determine differences between samples. In case variances were not equal, the Games-Howell test was performed^49^.

Three different glucosylated products were examined: multi-α-glucosylated product, containing residual RebA (RebA-G), mono-α-glucosylated product (RebA-G1) and RebA-G lacking RebA and RebA-G1 (RebA-G2+). Note that RebA-G, as defined here, contains a very minor amount of residual RebA, in contrast to the RebA-G as quantified by HPLC analysis.

### Data availability statement

All data generated or analyzed during this study are included in this published article (and its Supplementary Information files).

## Electronic supplementary material

Supplementary information
