# Supplementary material for: Glucansucrase (mutant) enzymes from Lactobacillus reuteri 180 efficiently transglucosylate Stevia component rebaudioside A, resulting in a superior taste
Source: Sci Rep. 2018 Jan 24;8:1516. doi: 10.1038/s41598-018-19622-5 (PMC5784128; doi:10.1038/s41598-018-19622-5)
Supplement: Supplementary file 1 — Supplementary information [file 41598_2018_19622_MOESM1_ESM.pdf]

Supplementary information belonging to

Glucansucrase (mutant) enzymes from *Lactobacillus reuteri* 180 efficiently transglucosylate *Stevia* component rebaudioside A, resulting in a superior taste

Evelien M. te Poele[a], Tim Devlamynck[a,b], Manuel Jäger[c], Gerrit J. Gerwig[a,d], Davy Van de Walle[e], Koen Dewettinck[e], Anna K. H. Hirsch[c], Johannis P. Kamerling[a,d], Wim Soetaert[b] & Lubbert Dijkhuizen[a]

[a]*Microbial Physiology, Groningen Biomolecular Sciences and Biotechnology Institute (GBB), University of Groningen, Nijenborgh 7, 9747 AG Groningen, The Netherlands*

[b]*Centre for Industrial Biotechnology and Biocatalysis, Department of Biochemical and Microbial Technology, Faculty of Bioscience Engineering, Ghent University, Coupure Links 653, 9000 Ghent, Belgium*

[c]*Stratingh Institute for Chemistry, University of Groningen, Nijenborgh 7, 9747 AG Groningen, The Netherlands*

[d]*NMR Spectroscopy, Bijvoet Center for Biomolecular Research, Utrecht University, Padualaan 8, 3584 CH Utrecht, The Netherlands*

[e]*Laboratory of Food Technology and Engineering, Faculty of Bioscience Engineering, Ghent University, Coupure Links 653, 9000 Ghent, Belgium*

Supplementary Table S1. Survey of wild-type glucansucrase Gtf180-ΔN of *L. reuteri* 180, and mutants derived, evaluated in this study. The 3-letter code DHT represents mutating amino acid residues D1085, R1088, and N1089 to D, H, and T, respectively. The same is valid for the other mutants shown with their 3-letter codes.

**mutation**	**feature**	**reference**
Gtf180-ΔN	wild-type; N-terminally truncated	31
Gtf180-ΔN-ΔV	domain V deletion mutant	32
PNS (V1027P:S1137N:A1139S)	triple mutant	35
Q1140E/A/H, S1137Y	near transition state stabilizing residue D1136	27
L940G/M/C/A/S/E/F/W	near acceptor subsite +1	34
L938A/S/F/K/M, A978F/S/G/L/P/Y, L981A/N/K, D1028Y/W/L/K/G/N, N1029Y/R/G/P/T/M	near acceptor subsite +1	25
DHT, NRL, VKG, YTS, ETL, AAA, MYM, FFF, DED, LLL, D1085Y/V/A/E/H/L/Q, R1088E/W/T/N/G/H/K, N1089Y/G/S/L/R/D/P	near acceptor subsite +2	33
W1065F/K/L/Q/G/T/E/F	near acceptor subsite +1 and +2	26
ΔV L938N	L938 mutation in Gtf180-ΔN-ΔV	unpublished
ΔV L940E/F	L940 mutations in Gtf180-ΔN-ΔV	32

Supplementary Table S2. Gtf180-ΔN mutants, screened for their RebA α-glucosylation potential with sucrose as donor substrate. Numbers shown here correspond to the TLC profiles (Supplementary Fig. S1). The 3-letter code DHT represents mutating amino acid residues D1085, R1088, and N1089 to D, H, and T, respectively. The same is valid for the other mutants shown with their 3-letter codes.

1. DHT	21. N1089D	41. D1085A	61. A978S
2. NRL	22. N1089P	42. D1085E	62. A978G
3. VKG	23. L940G	43. D1085H	63. A978L
4. YTS	24. L940M	44. D1085L	64. A978P
5. ETL	25. L940C	45. D1085Q	65. A978Y
6. AAA	26. L940A	46. R1088H	66. ΔV L938N
7. MYM	27. L940S	47. R1088K	67. ΔV L940E
8. FFF	28. L940E	48. D1028Y	68. ΔV L940F
9. DED	29. L940F	49. D1028W	69. W1065F
10. LLL	30. L940W	50. D1028L	70. W1065K
11. R1088E	31. L981A	51. D1028K	71. W1065L
12. R1088W	32. L981N	52. D1028G	72. W1065Q
13. R1088T	33. L981K	53. D1028N	73. W1065G
14. R1088N	34. L938A	54. N1029Y	74. W1065T
15. R1088G	35. L938S	55. N1029R	75. W1065E
16. N1089Y	36. L938F	56. N1029G	76. W1065F
17. N1089G	37. L938K	57. N1029P	77. Gtf180-ΔN
18. N1089S	38. L938M	58. N1029T	- no enzyme
19. N1089L	39. D1085Y	59. N1029M	
20. N1089R	40. D1085V	60. A978F	

Supplementary Table S3. Box-Behnken experimental design and results for the variables studied. Second-degree polynomial equation with coefficients of each factor is given for RebA conversion (%), RebA-G1/RebA-G (%) and amount of RebA-G synthesized (mM). RebA conversion = 85.4333 - 10.6000X_1 + 9.7500X_2 + 1.000X_3 - 5.8000X_1X_2 + 1.9000X_1X_3 + 1.1000X_2X_3 - 1.8667X_1^2 - 10.7667X_2^2 - 0.0667X_3^2. RebA-G1/RebA-G = 78.4333 – 0.2375X_1 – 3.5375X_2 + 1.5250X_3 + 2.1250X_1X_2 – 2.5500X_1X_3 – 0.8500X_2X_3 – 1.6292X_1^2 + 0.9708X_2^2 - 2.2542X_3^2. RebA-G = 106.8333 + 45.3250X_1 + 9.9750X_2 + 1.9750X_3 - 0.6750X_1X_2 + 2.7250X_1X_3 + 1.3250X_2X_3 – 10.3042X_1^2 – 12.3542X_2^2 – 1.1542X_3^2. X_1, RebA concentration (mM); X_2, D/A ratio; X_3, agitation speed (rpm).

	Pattern	X_1	X_2	X_3	RebA conversion (%)	RebA-G1/RebA-G (%)	RebA-G (mM)
**1**	+0+	200	2.5	200	73.8	76.3	147.6
**2**	0−+	125	1	200	64.3	83.4	80.6
**3**	−0+	50	2.5	200	94.1	77.2	47.1
**4**	0−−	125	1	0	63.4	80	79.3
**5**	0	200	4	100	66.6	74.8	133.2
**6**	−0−	50	2.5	0	97	67.7	48.6
**7**	−+0	50	4	100	96.5	75.8	48.3
**8**	0+−	125	4	0	82.7	72.6	103.4
**9**	−−0	50	1	100	67.4	85	33.8
**10**	0	125	2.5	100	85.5	78.9	106.9
**11**	+−0	200	1	100	60.7	75.6	121.4
**12**	0	125	2.5	100	85.4	78	106.8
**13**	0	125	2.5	100	85.4	78.5	106.8
**14**	0++	125	4	200	88	72.2	110
**15**	+0−	200	2.5	0	69.1	77	138.2

Supplementary Table S4. ^{1}H and ^{13}C chemical shifts (δ)[a] for the Glc*p* residues of RebA and RebA-G1, recorded in D_2O at 310 K. For structures, see Fig. 5 (main text).

Unit	RebA		RebA-G1		Unit	RebA		RebA-G1	
	^{1}H	^{13}C	^{1}H	^{13}C		^{1}H	^{13}C	^{1}H	^{13}C
**Glc1**(β1→C-19)					**Glc4**(β1→3)				
H-1	5.40	95.6	5.41	95.7	H-1	4.70	103.7	4.70	103.8
H-2	3.43	73.6	3.44	73.6	H-2	3.32	75.0	3.31	75.1
H-3	3.50	78.0	3.49	77.9	H-3	3.45	77.5	3.45	77.6
H-4	3.41	70.9	3.47	71.1	H-4	3.35	71.0	3.35	71.0
H-5	3.50	77.9	3.68	77.0	H-5	3.33	77.5	3.32	77.5
H-6a	3.82	62.3	3.87	*67.1*[b]	H-6a	3.85	62.9	3.85	62.8
H-6b	3.67		3.69		H-6b	3.65		3.66	
**Glc2**(β1→C-13)					**Glc5**(α1→6)				
H-1	4.71	97.6	4.70	97.5	H-1	-	-	4.86	99.4
H-2	3.68	*80.3*	3.67	*80.5*	H-2	-	-	3.47	73.1
H-3	3.81	*86.7*	3.82	*87.0*	H-3	-	-	3.66	74.9
H-4	3.46	70.4	3.46	70.5	H-4	-	-	3.35	71.0
H-5	3.45	77.5	3.44	77.5	H-5	-	-	3.63	73.3
H-6a	3.83	62.5	3.84	62.5	H-6a	-	-	3.75	62.1
H-6b	3.67		3.67		H-6b	-		3.65	
**Glc3**(β1→2)									
H-1	4.80	103.5	4.80	103.6					
H-2	3.21	76.0	3.20	75.9					
H-3	3.41	77.4	3.41	77.5					
H-4	3.26	71.9	3.25	72.0					
H-5	3.33	77.5	3.33	77.5					
H-6a	3.81	63.0	3.81	62.9					
H-6b	3.61		3.61						

[a] In ppm relative to the signal of internal acetone (δ 2.225 for ^{1}H and δ 31.07 for ^{13}C).

[b] Substituted carbon positions are indicated in italics.

Supplementary Table S5. Methylation analysis of the carbohydrate moieties in RebA and α-glucosylated RebA products RebA-G and RebA-G1. R_t, retention time relative to 1,5-di-*O*-acetyl-2,3,4,6-tetra-*O*-methylglucitol (1.00) on GLC; 2,3,4,6-Hex = 1,5-di-*O*-acetyl-2,3,4,6-tetra-*O*-methylglucitol-1-*d*. etc.; tr = trace (<2%).

Alditol acetate	R_t	Structural feature	Peak area (%)		
			RebA	RebA-G	RebA-G1
2,3,4,6-Hex	1.00	Glc*p* (1→	74	58	61
2,4,6-Hex	1.16	→3)Glc*p* (1→	-	3	-
3,4,6-Hex	1.18	→4)Glc*p* (1→	-	tr	tr
2,3,4-Hex	1.22	→6)Glc*p* (1→	-	17	18
4,6-Hex	1.32	→2,3)Glc*p* (1→	26	20	21
2,4-Hex	1.39	→3,6)Glc*p* (1→	-	2	-

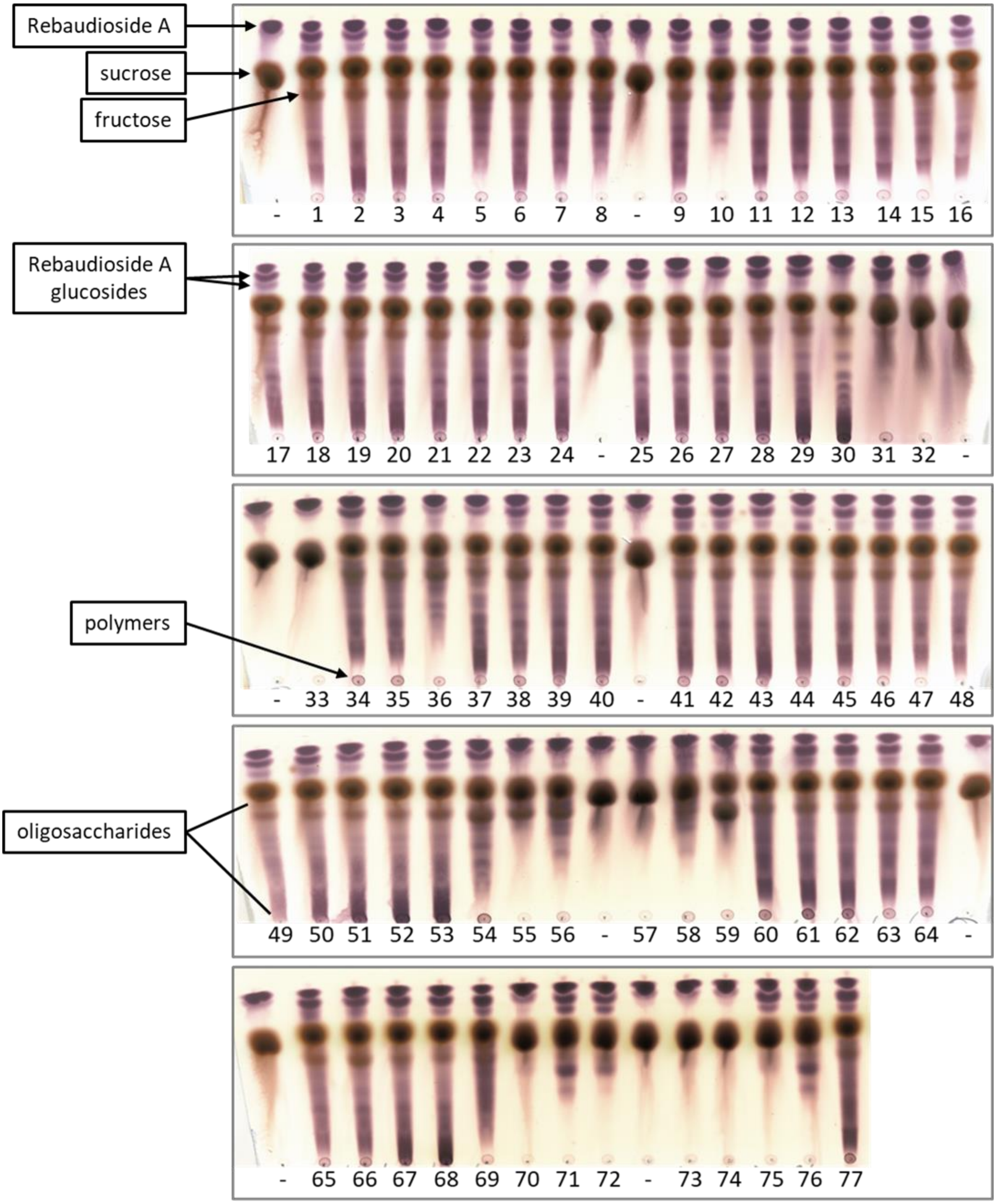

Supplementary Figure S1. TLC substrate/product profiles after a 3-h incubation at 37 °C of a buffer solution (pH 4.7) containing 50 mM RebA, 0.2 M sucrose and ~1 mg/mL wild-type Gtf180-ΔN (lane 77) or Gtf180-ΔN mutant enzymes (see Supplementary Table S1 and Table S2).

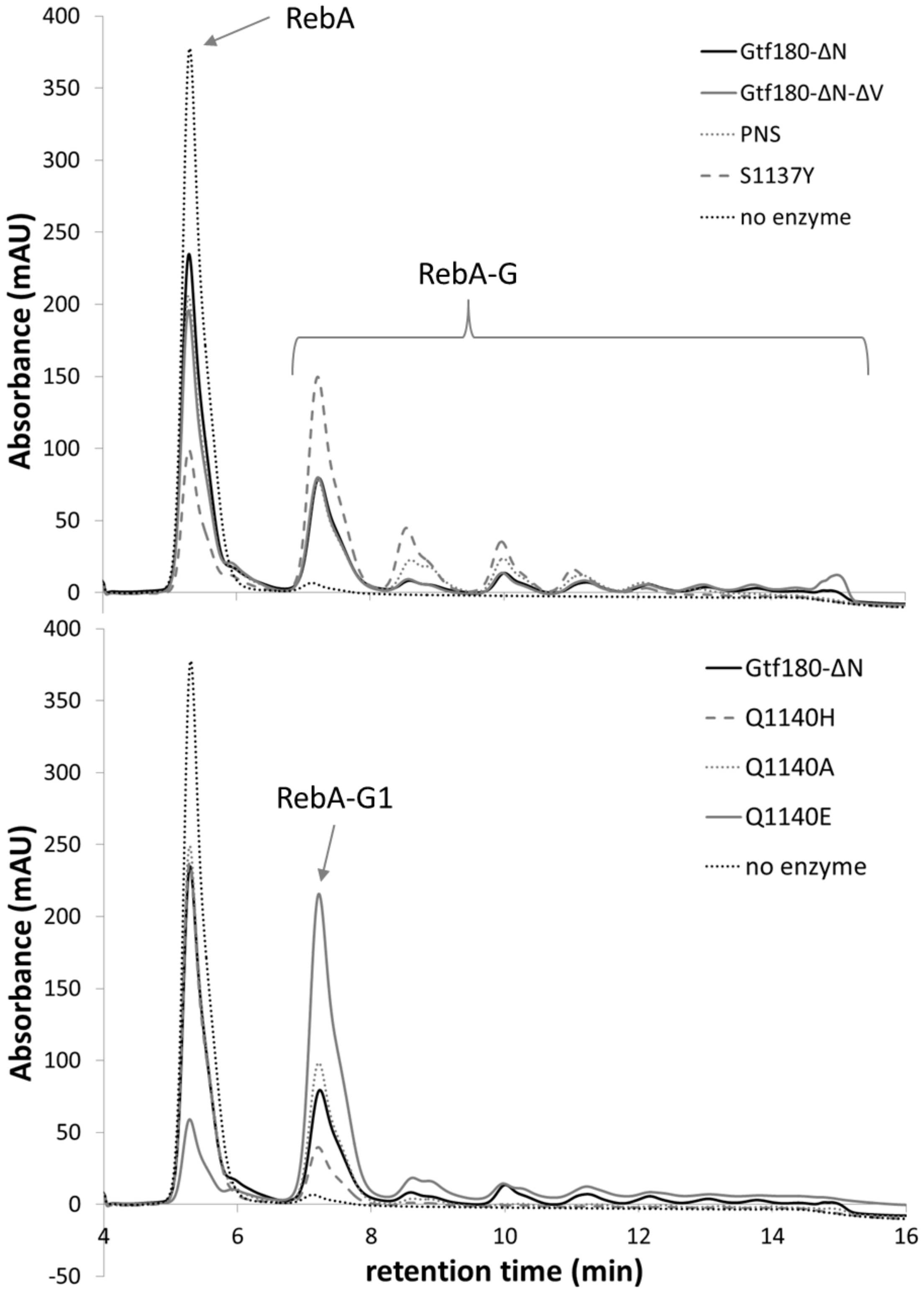

Supplementary Figure S2. HPLC substrate/product profiles after a 3-h incubation at 37 °C of a buffer solution (pH 4.7) containing 50 mM RebA, 1 M sucrose and ~1 mg/mL wild-type Gtf180-ΔN, wild-type Gtf180-ΔN-ΔV or Gtf180-ΔN mutant enzymes. RebA-G: total amount of α-glucosylated RebA product. RebA-G1: mono-α-glucosylated RebA.

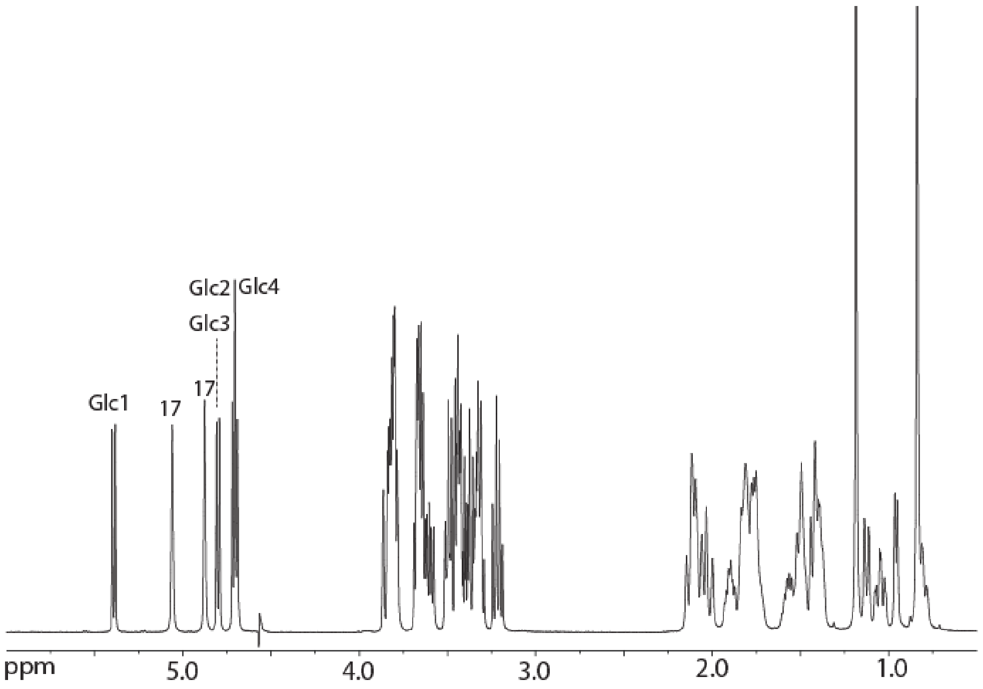

Supplementary Figure S3. 500-MHz ^{1}H NMR spectrum of commercial RebA, supplied by Tereos PureCircle, recorded in D_2O at 310K. The positions of the anomeric protons of the glucose residues (see Fig. 3) are indicated, as well as the steviol C-17 protons in the anomeric region. The spectrum is identical to that of commercial RebA, supplied by Aldrich-Sigma Chemie, and recently published with a complete assignment of resonances[23].

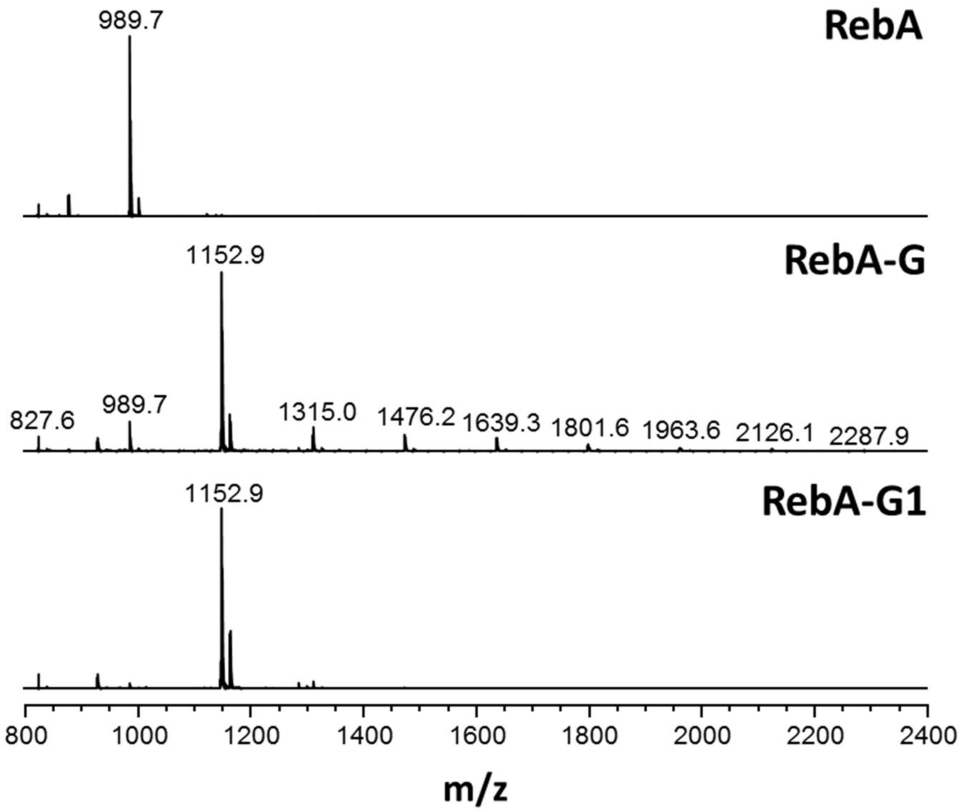

Supplementary Figure S4. MALDI-TOF mass spectra of RebA, RebA-G and RebA-G1.

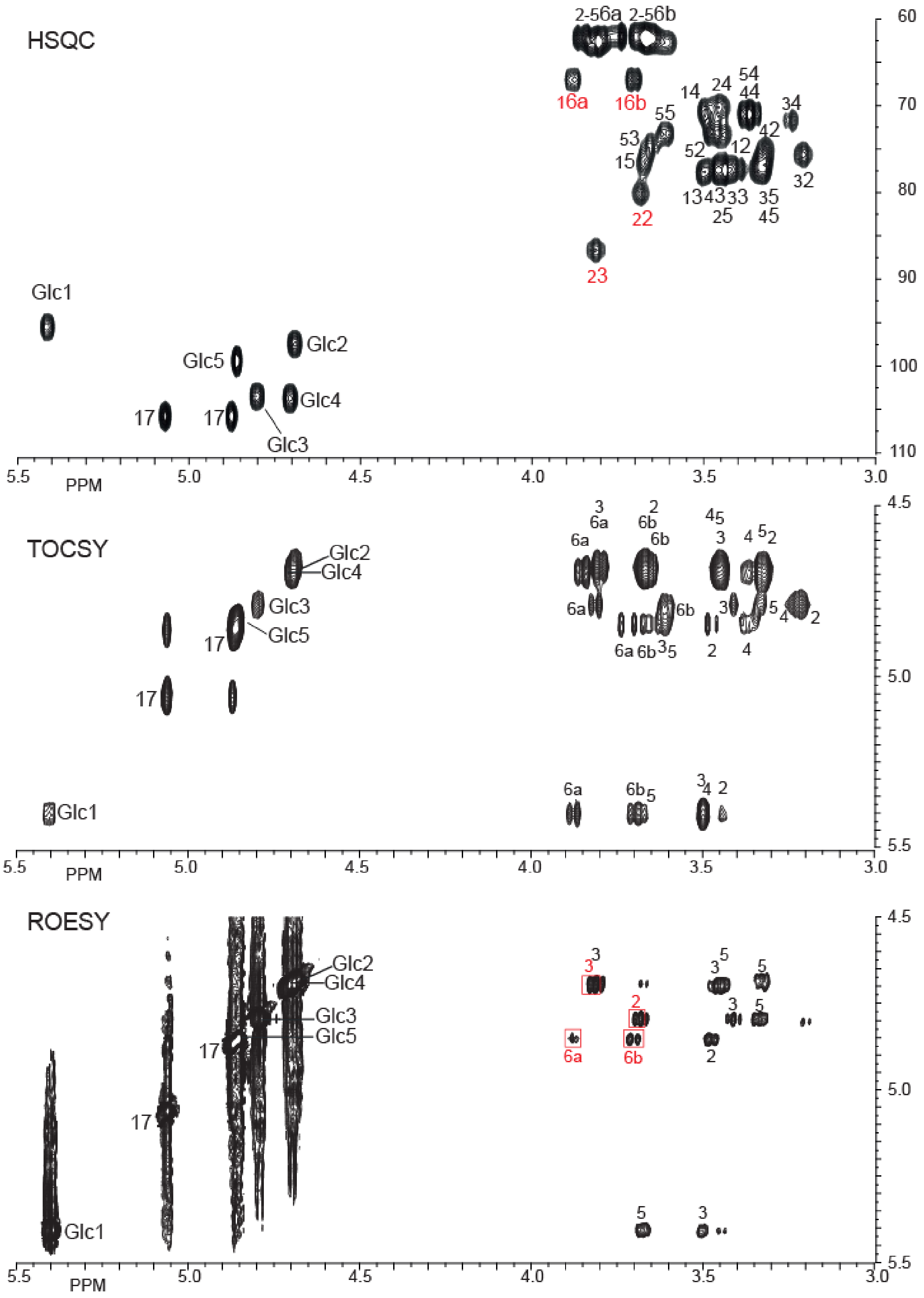

Supplementary Figure S5. HSQC, TOCSY (mixing time 200 ms) and ROESY spectra of the carbohydrate part of RebA-G1, recorded in D_2O at 310 K. In the HSQC spectrum, 23 means cross-peak H-3/C-3 of residue **Glc2**, etc.; assignments in red reflect the substituted positions of the residues. In the ROESY spectrum, the inter-residual cross-peaks confirming the **Glc4**(β1→3)**Glc2**, **Glc3**(β1→2)**Glc2** and **Glc5**(α1→6)**Glc1** linkages are indicated with red boxes.
